# Comparison of 2-year mortality according to obesity in stabilized patients with type 2 diabetes mellitus after acute myocardial infarction: results from the DIAMOND prospective cohort registry

**DOI:** 10.1186/s12933-015-0305-1

**Published:** 2015-10-15

**Authors:** Ki-Bum Won, Seung-Ho Hur, Yun-Kyeong Cho, Hyuck-Jun Yoon, Chang-Wook Nam, Kwon-Bae Kim, Jang-Ho Bae, Dong-Ju Choi, Young-Keun Ahn, Jong-Seon Park, Hyo-Soo Kim, Rak-Kyeong Choi, Donghoon Choi, Joon-Hong Kim, Kyoo-Rok Han, Hun-Sik Park, So-Yeon Choi, Jung-Han Yoon, Hyeon-Cheol Kwon, Seung-Un Rha, Kyung-Kuk Hwang, Do-Sun Lim, Kyung-Tae Jung, Seok-Kyu Oh, Jae-Hwan Lee, Eun-Seok Shin, Kee-Sik Kim

**Affiliations:** Division of Cardiology, Keimyung University Dongsan Medical Center, Daegu, South Korea; Division of Cardiology, Konyang University Hospital, Daejeon, South Korea; Division of Cardiology, Seoul National University Bundang Hospital, Seongnam, South Korea; Division of Cardiology, Chonnam National University Hospital, Gwangju, South Korea; Division of Cardiology, Yeungnam University Hospital, Daegu, South Korea; Division of Cardiology, Seoul National University Hospital, 28 Yeongeon-dong, Chongno-gu, Seoul, 110-744 South Korea; Division of Cardiology, Sejong General Hospital, Bucheon, South Korea; Division of Cardiology, Yonsei University Severance Hospital, Seoul, South Korea; Division of Cardiology, Pusan National University Yangsan Hospital, Yangsan, South Korea; Division of Cardiology, Hallym University Kangdong Sacred Heart Hospital, Seoul, South Korea; Division of Cardiology, Kyungpook National University Hospital, Daegu, South Korea; Division of Cardiology, Ajou University Hospital, Suwon, South Korea; Division of Cardiology, Wonju Severance Christian Hospital, Wonju, South Korea; Division of Cardiology, Samsung Medical Center, Seoul, South Korea; Division of Cardiology, Korea University Guro Hospital, Seoul, South Korea; Division of Cardiology, Chungbuk National University Hospital, Cheongju, South Korea; Division of Cardiology, Korea University Anam Hospital, Seoul, South Korea; Division of Cardiology, Eulji University Hospital, Daejeon, South Korea; Division of Cardiology, Wonkwang University Hospital, Iksan, South Korea; Division of Cardiology, Chungnam National University Hospital, Daejeon, South Korea; Division of Cardiology, Ulsan University Hospital, Ulsan, South Korea; Division of Cardiology, Daegu Catholic University Medical Center, Daegu, South Korea

**Keywords:** Type 2 diabetes mellitus, Acute myocardial infarction, Obesity, Survival

## Abstract

**Background:**

After acute myocardial infarction (AMI), the replicated phenomenon of obesity paradox, i.e., obesity appearing to be associated with increased survival, has not been evaluated in stabilized (i.e., without clinical events within 1 month post AMI) Asian patients with diabetes mellitus (DM).

**Methods:**

Among 1192 patients in the DIabetic Acute Myocardial InfarctiON Disease (DIAMOND) Korean multicenter registry between April 2010 and June 2012, 2-year cardiac and all-cause death were compared according to obesity (body mass index ≥25 kg/m^2^) in 1125 stabilized DM patients.

**Results:**

Compared with non-obese DM patients (62 % of AMI patients), obese DM patients had: higher incidence of dyslipidemia (31 vs. 24 %, P < 0.01); lower incidence of chronic kidney disease (26 vs. 33 %) (P < 0.01); higher left ventricular ejection fraction after AMI (53 ± 11 vs. 50 ± 12 %, P < 0.001); and lower 2-year cardiac and all-cause death occurrence (0.7 vs. 3.6 % and 1.9 vs. 5.2 %, both P < 0.01) and cumulative incidence in Kaplan–Meier analysis (P < 0.005, respectively). Likewise, both univariate and multivariate Cox hazard regression analyses adjusted for the respective confounders showed that obesity was associated with decreased risk of both cardiac [HR, 0.18 (95 % CI 0.06–0.60), P = 0.005; and 0.24 (0.07–0.78), P = 0.018, respectively] and all-cause death [0.34 (0.16–0.73), P = 0.005; and 0.44 (0.20–0.95), P = 0.038].

**Conclusions:**

In a Korean population of stabilized DM patients after AMI, non-obese patients appear to have higher cardiac and all-cause mortality compared with obese patients after adjusting for confounding factors.

**Electronic supplementary material:**

The online version of this article (doi:10.1186/s12933-015-0305-1) contains supplementary material, which is available to authorized users.

## Background

Obesity is strongly associated with an increased risk of numerous comorbidities and mortality in the general population [1–3]. In particular, obese subjects are more prone to be affected by cardiovascular (CV) events than non-obese subjects [4]. However, obesity appearing to be associated with improved survival has been reported after major CV events such as acute myocardial infarction (AMI) [5–9].

It is well established that diabetes mellitus (DM) is a major risk factor for CV morbidity and mortality [10, 11]. Previous studies have reported that diabetic patients have an increased risk for mortality after AMI [12, 13]. However, there is a paucity of data on the association between obesity and mortality in diabetic patients after the event of AMI; identifying this association may be more important in the Asian population because of the explicitly different features of DM in Asia [14–16]. In addition, it is necessary to evaluate this association in stabilized (i.e., without clinical events within 1 month) patients after AMI considering that it is difficult to identify the individual impact of clinical factors on early-term events after AMI [17]. Thus, we evaluated the association between obesity and 2-year mortality in stabilized diabetic patients after AMI in the Korean population.

## Methods

### Subjects and study design

This is a prospective, multicenter, observational study of clinical outcomes following AMI in patients with type 2 DM included in the DIabetic Acute Myocardial InfarctiON Disease (DIAMOND) registry in Korea between April 2010 and June 2012. Initially, 1192 consecutive patients with type 2 DM who presented with ST-elevation myocardial infarction (STEMI) or non ST-elevation myocardial infarction (NSTEMI) were enrolled from 22 university or tertiary hospitals that voluntarily participated in this study and were evenly distributed throughout South Korea. All participants had: (a) age ≥45 years; (b) documented STEMI or NSTEMI by an elevated creatine kinase-MB fraction (CK-MB) (exceeding 3 times upper limit of normal) and cardiac troponin-I level (exceeding upper normal limit); and (c) angiographically confirmed significant coronary stenosis (≥50 % luminal stenosis) with intracoronary filling defect or haziness suggesting coronary thrombus/vulnerable plaque, or coronary spasm induced acute myocardial infarction defined by an elevated cardiac enzymes without significant stenosis. Type 2 DM was diagnosed by fasting plasma glucose level on two separate occasions ≥126 mg/dL or a random plasma glucose level ≥200 mg/dL or 2-h plasma glucose post 75 g dextrose load done on two separate occasions ≥200 mg/dL, or previously diagnosed DM by taking oral hypoglycemic agents or using insulin. The duration of DM was defined as the time elapsed since a physician diagnosed the diabetic condition. BMI was calculated as weight (kg)/height (m^2^), and obesity was defined as a BMI of ≥25 kg/m^2^. Stabilized patients with AMI were defined as those who did not have any clinical events within 1 month after the initial presentation of AMI. Among these patients, 67 patients who died in hospital during admission or did not undergo follow-up within 1 month after discharge were excluded from the present study. Finally, 1125 diabetic patients with AMI who did not have any events within 1 month were enrolled for evaluation of cardiac and all-cause mortality according to obesity status.

Percutaneous coronary intervention (PCI) was performed by stenting (stent type and glycoprotein IIb/IIIa receptor blocker use at operator’s discretion) using standard technique via femoral or radial approach after a loading dose of aspirin 100–200 mg and clopidogrel 300–600 mg followed by a daily dose of aspirin 100 mg and clopidogrel 75 mg, and an intravenous bolus dose of heparin (50–100 U/kg) and thereafter 100 U/kg as needed to maintain an activated clotting time of >250 s during PCI. After the index procedure, aspirin 100–200 mg and clopidogrel 75 mg daily were prescribed for at least 12 months in patients treated with drug-eluting stent, as possible. All patients continued taking beta-blockers, angiotensin-converting enzyme inhibitors (ACEI) or angiotensin receptor blockers (ARB), and statins, whenever it was not contraindicated.

Coronary lesion morphology was classified using modified American College of Cardiology/American Heart Association criteria [18]. Thrombolysis in myocardial infarction (TIMI) score was used to determine the degree of coronary flow before and after the procedure [19]. Multivessel disease was defined as the presence of other lesions with ≥50 % stenosis in the non-infarct related coronary artery. Hypertension was defined as systolic blood pressure ≥140 mmHg and/or diastolic blood pressure ≥90 mmHg or treatment with antihypertensive agents. Dyslipidemia was defined as total cholesterol ≥240 mg/dL, low-density lipoprotein cholesterol (LDL) ≥130 mg/dL, high-density lipoprotein cholesterol (HDL) ≤40 mg/dL, triglyceride ≥150 mg/dL and/or treatment with lipid lowering agents. Transthoracic echocardiography was performed to assess the left ventricular ejection fraction (LVEF) using the modified Simpson’s bi-planar method. Chronic kidney disease (CKD) was defined as an estimated glomerular filtration rate (eGFR) <60 mL/min/1.73 m^2^ calculated by means of the modification of diet in renal disease formula [20]. The study outcomes were the occurrence of cardiac and all-cause death during 2-year follow-up. All death was considered cardiac unless there was a clear non-cardiac cause. According to the follow-up protocol, all patients were contacted at 1, 6, 12, and 24 months after the index procedures; if the patient did not attend a scheduled visit, outcome variables were obtained by telephone. The protocol of this study was approved by the appropriate Institutional Review Board/Ethical Committee of the respective clinical site, and informed consent was obtained from all participants.

### Statistical analysis

Clinical and biochemical characteristics are shown according to the presence of obesity. Values are expressed as mean ± SD for continuous variables and numbers and percentages for categorical variables. Continuous variables were compared using Student’s t test, and categorical variables were compared using the χ^2^ test or Fisher’s exact test, as appropriate. Kaplan–Meier survival analysis was performed for the cumulative occurrence of cardiac death and all-cause death. Comparisons between groups were performed using the log-rank test. Univariate and multivariate Cox hazard regression analyses were performed to identify the association between obesity and cardiac and all-cause death. Variables entered into the univariate analysis were selected focusing on traditional CV risk factors, procedural factors, the control status of hyperglycemia before AMI, and established clinical factors for mortality after AMI. Thus, univariate analysis included the following variables: old age (≥65 years), male gender, previous MI, hypertension, dyslipidemia, multivessel disease, stent diameter ≤2.75 mm, stent length ≥28 mm, HbA1c, CKD, LVEF <35 %, and obesity. Variables with P < 0.1 in the univariate analysis were entered into the multivariate Cox hazard regression analysis. The assumption of proportional hazards for the covariates included in the regression models was constant regardless of time, without significant interaction among them. SPSS statistical software version 20.0 (SPSS, Inc., Chicago, IL, USA) was used for all statistical analyses. Values of P < 0.05 were considered statistically significant.

## Results

The clinical characteristics of the 1125 participants (age, 65 ± 10 years; 66 % men) in this study are presented in Table 1. Overall, the mean duration of DM was 10.9 ± 8.5 years, mean hemoglobin A1c (HbA1c) level was 7.8 ± 1.5 %, and mean BMI was 24.1 ± 3.0 kg/m^2^ in the present study. The prevalence of obesity was 38 %, and the majority of participants therefore were non-obese. The incidence of BMI <18.5 kg/m^2^ and BMI ≥30 kg/m^2^ was only 2.2 and 3.6 %, respectively.

**Table 1:** Clinical characteristics

	Obesity (n = 427)	Non-obesity (n = 698)	P
Age, years	63 ± 10	66 ± 10	<0.001
Male	283 (66)	457 (66)	0.783
BMI, kg/m^2^	27.1 ± 2.0	22.3 ± 1.8	<0.001
Co-existing conditions			
Hypertension	294 (69)	448 (64)	0.109
Dyslipidemia	134 (31)	169 (24)	0.009
CKD	110 (26)	232 (33)	0.008
Previous MI	22 (5)	39 (6)	0.754
Smoking	143 (34)	229 (33)	0.814
STEMI	204 (48)	326 (47)	0.727
LVEF	53 ± 11	50 ± 12	<0.001
Systolic blood pressure, mmHg	130 ± 28	130 ± 28	0.851
Diastolic blood pressure, mmHg	78 ± 17	76 ± 16	0.116
eGFR, mL/min/1.73 m^2^	76 ± 29	72 ± 33	0.105
DM duration, years	9.8 ± 8.2	11.5 ± 8.5	0.003
Laboratory			
Total cholesterol, mg/dL	178 ± 46	170 ± 46	0.004
Triglyceride, mg/dL	146 ± 98	131 ± 100	0.022
LDL, mg/dL	109 ± 40	101 ± 41	0.002
HDL, mg/dL	44 ± 28	44 ± 17	0.988
Creatinine, mg/dL	1.2 ± 1.3	1.3 ± 1.4	0.130
HbA1c, %	7.8 ± 1.4	7.9 ± 1.6	0.370
hs-CRP, mg/dL	4.6 ± 15.9	6.9 ± 23.9	0.081
NT-ProBNP, pg/mL	2836 ± 7592	4040 ± 9074	0.096
Peak CK-MB, ng/mL	82 ± 114	83 ± 137	0.880
Troponin-I, ng/mL	29 ± 62	31 ± 59	0.637
Medication at discharge, n (%)			
Aspirin	423 (99)	683 (98)	0.126
Clopidogrel	409 (96)	661 (95)	0.413
Cilostazol	83 (19)	131 (19)	0.781
Beta blocker	364 (85)	591 (85)	0.794
ACEI/ARB	355 (83)	586 (84)	0.720
Statin	365 (86)	572 (82)	0.123
Nitrate	111 (26)	205 (29)	0.222
Nicorandil	77 (18)	149 (21)	0.178
Insulin	51 (12)	119 (17)	0.020
2-year clinical outcomes, n (%)			
Cardiac death	3 (0.7)	25 (3.6)	0.003
All-cause death	8 (1.9)	36 (5.2)	0.006

*ACE-I* angiotensin converting enzyme inhibitor, *ARB* angiotensin receptor blocker, *BMI* body mass index, *CKD* chronic kidney disease, *CK-MB* creatine kinase-MB, *DM* diabetes mellitus, *eGFR* estimated glomerular filtration rate, *HbA1c* hemoglobin A1c, *HDL* high density lipoprotein, *hs-CRP* high sensitivity C-reactive protein, *LDL* low density lipoprotein, *LVEF* left ventricular ejection fraction, *MI* myocardial infarction, *STEMI* ST-elevation MI

The distributions of baseline characteristics did not differ between obese and non-obese diabetics, except for those of age, duration of DM, and the incidence of CKD and insulin use which were significantly higher in non-obese DM patients, while obese DM patients had higher incidence of dyslipidemia and higher LVEF after AMI (Table 1). As shown in Table 2, there were no significant differences in angiographic and procedural characteristics, except for significantly larger stent diameter in obese vs. non-obese DM patients (3.18 ± 0.46 vs 3.07 ± 0.43 mm, P < 0.001).

**Table 2:** Angiographic and procedural characteristics

	Obesity (n = 427)	Non-obesity (n = 698)	P
Target vessel of LAD	209 (49)	355 (51)	0.533
Target vessel of LM	11 (3)	17 (2)	0.883
Multivessel disease	254 (60)	414 (59)	0.954
Type B2/C lesion	340 (82)	543 (83)	0.850
Pre-PCI TIMI 0	173 (42)	260 (40)	0.465
Post-PCI TIMI 2/3	400 (97)	639 (97)	0.592
Use of DES	341 (93)	540 (93)	0.684
Stent diameter, mm	3.18 ± 0.46	3.07 ± 0.43	<0.001
Stent length, mm	25.4 ± 9.6	24.8 ± 7.9	0.324
Number of implanted stents	1.6 ± 0.9	1.5 ± 0.8	0.703

*DES* drug-eluting stent, *LAD* left anterior descending artery, *LM* left main coronary artery, *TIMI* thrombolysis in myocardial infarction

**Fig. 1:**
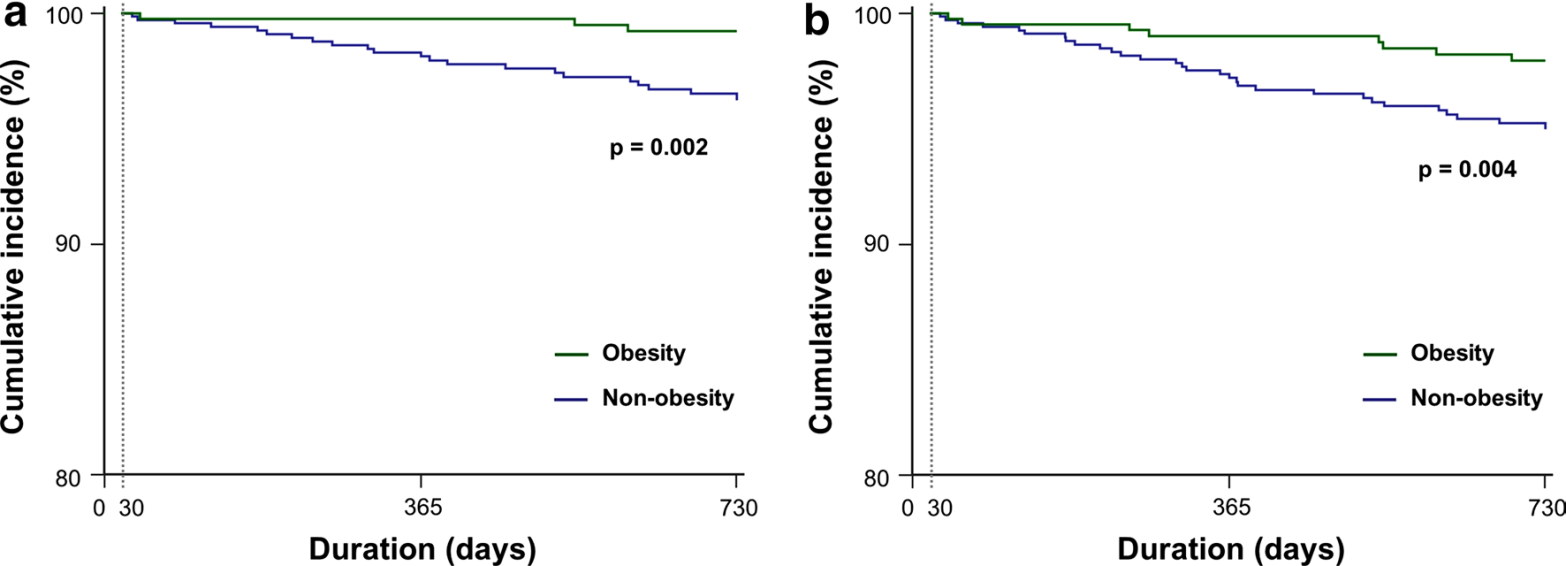
Kaplan–Meier analysis of **a** cumulative cardiac death-free survival and **b** cumulative all-cause death-free survival according to obesity presence among all participants

During 2-year follow-up period, a total of 28 cardiac deaths and 44 all-cause deaths occurred. The occurrence of cardiac death and all-cause death was significantly lower in diabetic patients with than without obesity (cardiac death: 0.7 vs. 3.6 %, P = 0.003; all-cause death: 1.9 vs. 5.2 %, P = 0.006) (Table 1). Kaplan–Meier survival analysis revealed that the cumulative incidence of cardiac death (P = 0.002) and all-cause death (P = 0.004) was lower in diabetic patients with obesity than in those without obesity (Fig. 1a, b). Kaplan–Meier survival analysis revealed consistent result after excluding underweight patients with BMI <18.5 kg/m^2^ (Additional file 1: Figure S1, A and B).

Cox hazard regression models were performed to identify the determinants of cardiac and all-cause death in stabilized DM patients (Table 3). Both univariate and multivariate Cox hazard regression analyses adjusted for the respective confounders showed that obesity was associated with decreased risk of both cardiac [HR, 0.18 (95 % CI 0.06–0.60), P = 0.005; and 0.24 (0.07–0.78), P = 0.018, respectively] and all-cause death [0.34 (0.16–0.73), P = 0.005; and 0.44 (0.20–0.95), P = 0.038]. In terms of confounders, in univariate Cox hazard regression analysis, age ≥65 years, CKD, and LVEF <35 % were significantly associated with increased risk of cardiac and all-cause death, while previous MI was associated with cardiac death. In multivariate Cox hazard regression analysis, CKD and LVEF <35 % were associated with increased risk of cardiac and all-cause death, and age ≥65 years with all-cause death.

**Table 3:** Cox hazard regression models to identify the independent determinants for cardiac and all-cause death

	Cardiac death				All-cause death			
	Univariate		Multivariate		Univariate		Multivariate	
	HR (95 % CI)	P	HR (95 % CI)	P	HR (95 % CI)	P	HR (95 % CI)	P
Age ≥65 years	4.52 (1.72–11.88)	0.002	2.65 (0.98–7.15)	0.055	3.99 (1.91–8.31)	<0.001	2.58 (1.09–6.10)	0.031
Male	0.68 (0.32–1.44)	0.317			0.56 (0.31–1.02)	0.056	0.63 (0.34–1.19)	0.157
Previous MI	3.07 (1.07–8.85)	0.038	2.37 (0.81–6.94)	0.114	2.41 (0.95–6.11)	0.065	1.80 (0.70–4.63)	0.227
Hypertension	1.94 (0.79–4.79)	0.150			1.98 (0.95–4.13)	0.069	1.35 (0.60–3.00)	0.466
Dyslipidemia	1.33 (0.60–2.94)	0.481			1.21 (0.63–2.32)	0.563		
CKD	3.62 (1.70–7.73)	0.001	2.47 (1.11–5.54)	0.028	4.38 (2.34–8.21)	<0.001	3.10 (1.56–6.17)	0.001
Multivessel disease	1.19 (0.55–2.57)	0.663			1.08 (0.59–1.99)	0.801		
HbA1c, %	1.17 (0.90–1.52)	0.245			1.01 (0.80–1.29)	0.920		
Stent diameter ≤2.75 mm	0.87 (0.28–2.75)	0.817			0.71 (0.29–1.76)	0.456		
Stent length ≥28 mm	1.62 (0.59–4.49)	0.350			1.86 (0.88–3.95)	0.105		
LVEF <35 %	6.09 (2.79–13.30)	<0.001	4.18 (1.90–9.23)	<0.001	4.77 (2.47–9.21)	<0.001	3.35 (1.72–6.53)	<0.001
Obesity	0.18 (0.06–0.60)	0.005	0.24 (0.07–0.78)	0.018	0.34 (0.16–0.73)	0.005	0.44 (0.20–0.95)	0.038

*CI* confidence interval, *CKD* chronic kidney disease, *HbA1c* hemoglobin A1c, *HR* hazard ratio, *LVEF* left ventricular ejection fraction, *MI* myocardial infarction

## Discussion

The main findings of the present analysis of the DIAMOND registry investigating the long-term clinical outcomes after contemporary treatment in stabilized diabetic patients with AMI in the Korean population are: (1) the majority of Korean diabetic patients with AMI are non-obese; (2) obesity is associated with decreased cardiac and all-cause mortality after adjusting for confounding clinical factors.

It is well known that obesity is strongly associated with an increased risk of CV mortality. Song et al. [21] reported that higher CV mortality was observed in men compared with women across categories of anthropometric measures of obesity, and the gender difference was attenuated in obese subjects. Novo et al. [22] reported that subclinical atherosclerosis, especially if it was associated with metabolic syndrome (MetS) which has central obesity as its major characteristic, leads to an increased risk of CV mortality. These studies were performed in the general population without consideration of major CV events such as AMI. In contrast, several recent studies reported the phenomenon of obesity paradox, i.e. long-term mortality appearing to be significantly lower among obese than non-obese patients after the event of AMI, albeit with different independent predictive value of obesity for long-term mortality after adjusting for confounding factors [23, 24]. There is a paucity of data on whether this phenomenon is observed in patients with DM, which is considered a coronary artery disease risk-equivalent in clinical practice [25].

Both a deterioration of insulin secretion and an aggravation of insulin resistance are two pivotal defects in the pathogenesis of DM [26, 27]. It is obvious that obesity is one of the major factors for insulin resistance, but the criterion and prevalence of obesity differ according to ethnicity. Moreover, the clinical features of the development of type 2 DM in Asia are somewhat different from those in other parts of the world, with DM developing at a younger age and in subjects with much lower BMI despite the substantial increases in the prevalence of obesity and type 2 DM in Asia [14]. In Korea, previous studies reported that 65 % of diabetic subjects are non-obese and that impaired insulin secretion is more prominent than insulin resistance in the pathogenesis of type 2 DM, even in the status of impaired glucose tolerance [28–30]. In the present study, the majority of diabetic patients, approximately 62 %, also was non-obese. This value is very similar compared to the previous studies although our study was performed in diabetic patients after the event of AMI.

Although the phenomenon of the obesity paradox, i.e., obesity appearing to be associated with improved survival in patients with AMI, has been replicated [5–9], there is a paucity of data on the association between obesity and CV mortality in diabetic patients with AMI, and it is uncertain whether this effect persists in stabilized patients after the event of AMI, which warrants investigation because the confounding relationships among multiple clinical factors may be able to simultaneously influence development of early-term events [17]. A recent study reported a strong protective effect of overweight or obesity on all-cause mortality in AMI patients without DM, but the effect was not found among those with DM [31]. However, in the latter German population-based AMI registry study the prevalence of overweight or obesity defined as a BMI ≥25 kg/m^2^ was up to 81 % among diabetic patients. In particular, the prevalence of obesity defined as a BMI ≥30 kg/m^2^ was almost 38 % among diabetic patients. In contrast, the prevalence of BMI ≥30 kg/m^2^ was only 3.6 % (41 patients) in the present study, which might have contributed to the different results obtained.

The exact mechanism by which obesity improves survival after the event of AMI is unknown. However, one potential explanation is that obese patients may have less severe left ventricular systolic dysfunction after the event of AMI. In experimental data, using a diet-induced obesity model, Poncelas et al. [32] suggested the beneficial effect of increased insulin signaling as the mechanism underlying the obesity paradox, and Salie et al. [33] reported that obesity-inducing diets appeared to have a cardioprotective effect against ischemia or reperfusion damage. In clinical data, Lundergan et al. [34] reported that high BMI was associated with an increased effect of preservation of LVEF and improved 30-day survival in patients with AMI. Sohn et al. [35] recently reported that obesity is independently associated with smaller infarct size which was identified using contrast-enhanced magnetic resonance imaging in Korean patients undergoing primary PCI for STEMI. In the present study, diabetic patients with obesity had significantly higher LVEF compared to those without obesity after AMI. In addition, the incidence of LVEF <35 % that was independently associated with both cardiac and all-cause death was significantly lower among diabetic patients with than those without obesity.

Several previous studies investigated the association between obesity and coronary atherosclerosis using coronary computed tomographic angiography. Labounty et al. [36] reported that an increased BMI was associated with a greater prevalence, extent, and severity of coronary artery disease (CAD). Dores et al. [37] reported that obesity was associated with the presence of CAD, but it was not correlated with the severity of CAD in subjects with suspected CAD. However, these studies were based on Western populations and evaluated the relationship between BMI and coronary atherosclerosis without the consideration of diabetic status. Recently, Won et al. [38] reported that DM was strongly associated with coronary parameters including any plaque, obstructive plaque, and coronary artery calcium score (CACS) >100 in a Korean population. According to this study, the prevalence of obesity was significantly higher in diabetic subjects than in non-diabetic subjects but the majority of diabetic patients were non-obese. Obesity was independently associated with the presence of any plaque and CACS >100 in non-diabetic subjects, but it was not associated with any coronary parameters in established diabetic subjects. These results may imply that obesity contributes to the development of DM but it is not a useful predictor for the presence and severity of CAD in established diabetic subjects in a Korean population. Further investigation to identify the association between obesity and major CV complications may be necessary in Asian diabetic patients.

The prevalence of obesity which is significantly associated with dyslipidemia, type 2 DM, and CV disease has increased worldwide. Although BMI is the most commonly used anthropometric tool to assess obesity status, BMI which might not be the ideal measure to discriminate between fat and lean body mass. Considering that recent studies emphasized the quality or function of adipose tissue compared with its amount with respect to CV disease [39] and the optimal treatment for atherogenic dyslipidemia in association with obesity [40], it is necessary to evaluate the phenomenon of obesity paradox focusing on these issues. Additionally, further study is warranted to assess whether the phenomenon of obesity paradox results from a statistical artifact associated with collider stratification bias [41].

The present study has some limitations. First, we identified an association between obesity and survival but did not present the range of BMI over which it held, which was difficult to determine because only 41 (3.6 %) of our diabetic patients had a BMI ≥30 kg/m^2^. The latter proportion was very small compared to those reported in other studies investigating the obesity paradox phenomenon, which might be related to the explicitly different characteristics of DM in the Asian population. Second, we only used BMI which might not be the ideal measure to discriminate between fat and lean body mass to identify obesity status; however, a previous study reported that BMI was significantly associated with abdominal fat and waist circumference in Korean subjects [42]. Third, the present study might have underestimated the risk of mortality because clinical events are likely to occur in the acute stage after AMI presentation, especially for high-risk patients. However, this study was performed to focus on excluding confounding impact of multiple factors during the early stage after the event of AMI. Finally, we did not control for the stent type which may have influenced study results. Despite its limitations, the present study is unique in that it identified the obesity paradox in stabilized diabetic patients after the event of AMI in an Asian population. Considering the different clinical features of diabetic patients in Asian compared to Western populations, the results of this study may provide valuable information on the association between obesity and prognosis after the event of AMI in Asian diabetic patients.

## Conclusion

In a Korean population of stabilized DM patients after the event of AMI, cardiac and all-cause mortality appeared to be higher in non-obese than obese patients. Obesity was significantly associated with decreased risk for cardiac and all-cause death after adjusting for confounding risk factors.

## Additional file

10.1186/s12933-015-0305-1 Kaplan–Meier analysis of (A) cumulative cardiac death-free survival and (B) cumulative all-cause death-free survival according to categorical BMI among all participants.

## Abbreviations

ACEI: angiotensin-converting enzyme inhibitors
AMI: acute myocardial infarction
ARB: angiotensin receptor blockers
BMI: body mass index
CACS: coronary artery calcium score
CAD: coronary artery disease
CI: confidence interval
CKD: chronic kidney disease
CK-MB: creatine kinase-MB
CV: cardiovascular
DES: drug-eluting stent
DM: diabetes mellitus
eGFR: estimated glomerular filtration rate
HbA1c: hemoglobin A1c
HDL: high-density lipoprotein
HR: hazard ratio
hs-CRP: high sensitivity C-reactive protein
LAD: left anterior descending artery
LDL: low-density lipoprotein
LM: left main coronary artery
LVEF: left ventricular ejection fraction
MetS: metabolic syndrome
MI: myocardial infarction
NSTEMI: non ST-elevation myocardial infarction
NT-ProBNP: N-terminal prohormone of brain natriuretic peptide
PCI: percutaneous coronary intervention
QCA: quantitative coronary angiography
STEMI: ST-elevation myocardial infarction
TIMI: thrombolysis in myocardial infarction
