# Correction: Comparison of 2‑year mortality according to obesity in stabilized patients with type 2 diabetes mellitus after acute myocardial infarction: results from the DIAMOND prospective cohort registry

**DOI:** 10.1186/s12933-023-01889-2

**Published:** 2023-06-29

**Authors:** Ki‑Bum Won, Seung‑Ho Hur, Yun‑Kyeong Cho, Hyuck‑Jun Yoon, Chang‑Wook Nam, Kwon‑Bae Kim, Jang‑Ho Bae, Dong‑Ju Choi, Young‑Keun Ahn, Jong‑Seon Park, Hyo‑Soo Kim, Rak‑Kyeong Choi, Donghoon Choi, Joon‑Hong Kim, Kyoo‑Rok Han, Hun‑Sik Park, So‑Yeon Choi, Jung‑Han Yoon, Hyeon‑Cheol Kwon, Seung-Woon Rha, Kyung‑Kuk Hwang, Do‑Sun Lim, Kyung‑Tae Jung, Seok‑Kyu Oh, Jae‑Hwan Lee, Eun‑Seok Shin, Kee‑Sik Kim

**Affiliations:** 1 Division of Cardiology, Keimyung University Dongsan Medical Center, Daegu, South Korea; 2 Division of Cardiology, Konyang University Hospital, Daejeon, South Korea; 3 Division of Cardiology, Seoul National University Bundang Hospital, Seongnam, South Korea; 4 Division of Cardiology, Chonnam National University Hospital, Gwangju, South Korea; 5 Division of Cardiology, Yeungnam University Hospital, Daegu, South Korea; 6 Division of Cardiology, Seoul National University Hospital, 28 Yeongeon-Dong, Chongno-Gu, Seoul, 110-744 South Korea; 7 Division of Cardiology, Sejong General Hospital, Bucheon, South Korea; 8 Division of Cardiology, Yonsei University Severance Hospital, Seoul, South Korea; 9 Division of Cardiology, Pusan National University Yangsan Hospital, Yangsan, South Korea; 10 Division of Cardiology, Hallym University Kangdong Sacred Heart Hospital, Seoul, South Korea; 11 Division of Cardiology, Kyungpook National University Hospital, Daegu, South Korea; 12 Division of Cardiology, Ajou University Hospital, Suwon, South Korea; 13 Division of Cardiology, Wonju Severance Christian Hospital, Wonju, South Korea; 14 Division of Cardiology, Samsung Medical Center, Seoul, South Korea; 15 Division of Cardiology, Korea University Guro Hospital, Seoul, South Korea; 16 Division of Cardiology, Chungbuk National University Hospital, Cheongju, South Korea; 17 Division of Cardiology, Korea University Anam Hospital, Seoul, South Korea; 18 Division of Cardiology, Eulji University Hospital, Daejeon, South Korea; 19 Division of Cardiology, Wonkwang University Hospital, Iksan, South Korea; 20 Division of Cardiology, Chungnam National University Hospital, Daejeon, South Korea; 21 Division of Cardiology, Ulsan University Hospital, Ulsan, South Korea; 22 Division of Cardiology, Daegu Catholic University Medical Center, Daegu, South Korea

**Correction: Cardiovasc Diabetol (2015) 14:141** 10.1186/s12933-015-0305-1

Following publication of the original article [1], the author name “Seung-Woon Rha” was incorrectly written as “Seung-Un Rha”. This has now been corrected with this erratum.
