# Supplementary figures and images for: Comparison of 2-year mortality according to obesity in stabilized patients with type 2 diabetes mellitus after acute myocardial infarction: results from the DIAMOND prospective cohort registry
Source: Cardiovasc Diabetol. 2015 Oct 15;14:141. doi: 10.1186/s12933-015-0305-1 (PMC4608118; doi:10.1186/s12933-015-0305-1)

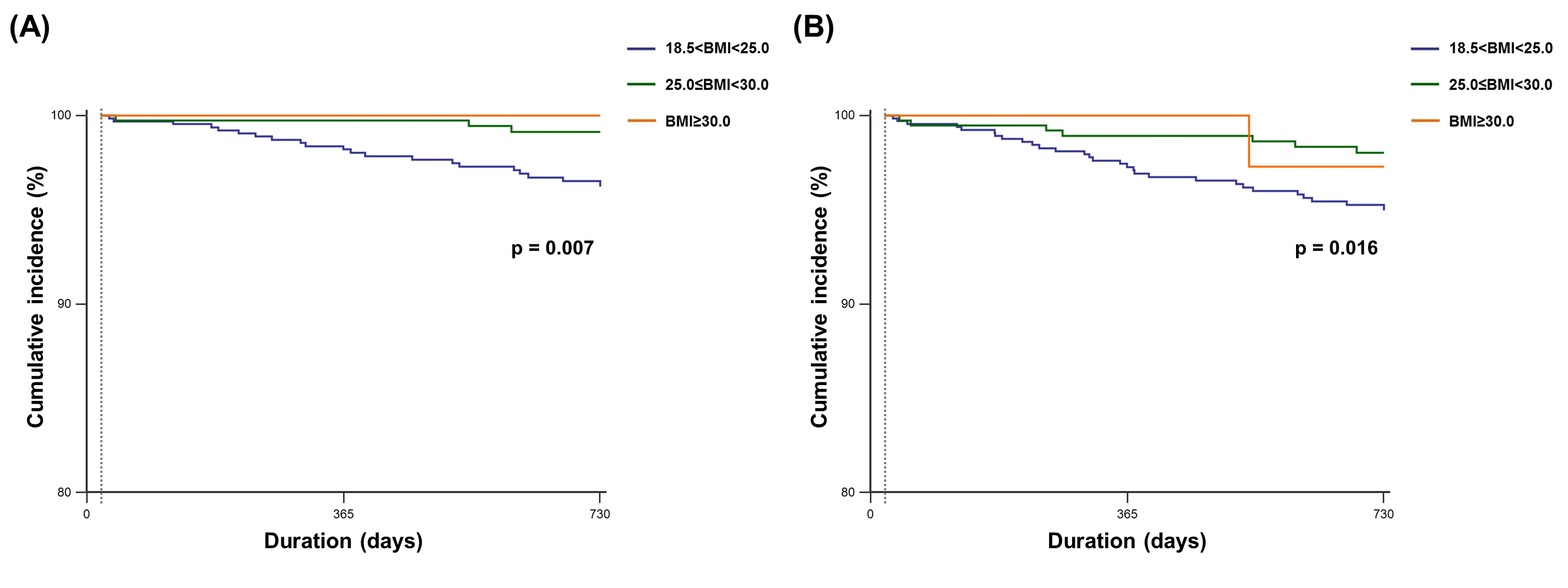

Supplement: Supplementary file 1 — 10.1186/s12933-015-0305-1 Kaplan–Meier analysis of (A) cumulative cardiac death-free survival and (B) cumulative all-cause death-free survival according to categorical BMI among all participants. [file 12933_2015_305_MOESM1_ESM.tif]
